# Effect of Intrapleural Fibrinolytic Therapy vs Surgery for Complicated Pleural Infections: A Randomized Clinical Trial

**DOI:** 10.1001/jamanetworkopen.2023.7799

**Published:** 2023-04-12

**Authors:** Candice L. Wilshire, Anee S. Jackson, Eric Vallières, Adam J. Bograd, Brian E. Louie, Ralph W. Aye, Alexander S. Farivar, Peter T. White, Christopher R. Gilbert, Jed A. Gorden

**Affiliations:** 1 Division of Thoracic Surgery and Interventional Pulmonology, Swedish Cancer Institute, Seattle, Washington

## Abstract

**Importance:**

There is a paucity of high-quality prospective randomized clinical trials comparing intrapleural fibrinolytic therapy (IPFT) with surgical decortication in patients with complicated pleural infections.

**Objective:**

To assess the feasibility, safety, and efficacy of an algorithm comparing tissue plasminogen activator plus deoxyribonuclease therapy with surgical decortication in patients with complicated pleural infections.

**Design, Setting, and Participants:**

This parallel pilot randomized clinical trial was performed at a single urban community-based center from March 1, 2019, to December 31, 2021, with follow-up for 90 days. Seventy-four individuals were screened and 48 were excluded. Twenty-six patients 18 years or older with clinical pleural infection and positive findings of pleural fluid analysis were included. Of these, 20 patients underwent randomized selection (10 in each group), and 6 were observed.

**Interventions:**

Intrapleural tissue plasminogen activator plus deoxyribonuclease therapy vs surgical decortication.

**Main Outcomes and Measures:**

Primary outcomes were the percentage of patients enrolled to study completion and multidisciplinary adherence. Secondary outcomes included the number of patients with and the reason for inadequate screening, screening to enrollment failures, time to accrual of 20 patients or the number accrued at 1 year, and clinical data.

**Results:**

Twenty-six patients were enrolled, 10 were randomized to each group, and 6 were observed. There was 100% enrollment to study completion in each treatment group, no protocol deviations, 2 minor protocol amendments, and no screening to enrollment failures. It took 32 months to enroll 26 patients. The 20 randomized patients had a median age of 57 (IQR, 46-65) years, were predominantly men (15 [75%]), and had a median RAPID (Renal, Age, Purulence, Infection Source, and Dietary Factors) score of 2 (IQR, 1-3). Treatment failure occurred in 1 patient and 2 crossover treatments occurred, all of which were in the IPFT group. Intraprocedure and postprocedure complications were similar between the groups. There were no reoperations or in-hospital deaths. Median duration of chest tube use was comparable in the IPFT (5 [IQR, 4-8] days) and surgery (4 [IQR, 3-5] days) groups (*P* = .21). Median hospital stay tended to be longer in the IPFT (11 [IQR, 4-18] days) vs surgery (5 [IQR, 4-6] days) groups, although the difference as not significantly different (*P* = .08). There were no 30-day readmissions or 30- or 90-day deaths.

**Conclusions and Relevance:**

In this pilot randomized clinical trial, the study algorithm was feasible, safe, and efficacious. This provides evidence to move forward with a multicenter randomized clinical trial.

**Trial Registration:**

ClinicalTrials.gov Identifier: NCT03873766

## Key Points

**Question:**

Is an algorithm comparing tissue plasminogen activator plus deoxyribonuclease therapy with surgical decortication in patients with complicated pleural infections feasible, safe, and efficacious?

**Findings:**

In this pilot randomized clinical trial of 20 participants, there was 100% enrollment to study completion, no protocol deviations, 2 minor protocol amendments, and no screen to enrollment failures.

**Meaning:**

The study algorithm was feasible, safe, and efficacious, providing evidence to move forward with a multicenter randomized clinical trial.

## Teaser

This randomized clinical trial compares chest tube drainage of the pleural cavity using intrapleural fibrinolytic therapy and surgical decortication among patients with complicated pleural infections.

## Introduction

Complicated pleural infections (CPIs), including complicated parapneumonic effusions and empyemas, are common, affecting approximately 80 000 patients each year in the US and United Kingdom combined.^1^ Management is challenging, as these infections are associated with significant morbidity and an all-cause mortality rate that may approach 18%.^2,3,4,5,6,7,8,9,10,11,12^ Hospital stays are prolonged at a mean of 14 days, and medical costs approach $40 000 per episode.^7,8,13,14^

Initial treatment of CPI requires antibiotic therapy and drainage; however, more aggressive management is often required. Next management steps include surgery or intrapleural fibrinolytic therapy (IPFT) with tissue plasminogen activator and deoxyribonuclease. There is a paucity of evidence directly comparing the efficacy and outcomes of IPFT management with surgery, and most published studies have significant methodological weaknesses. This lack of evidence leads to challenging decision-making for clinicians. Even though the Multicenter Intrapleural Sepsis Trial (MIST II) demonstrated IPFT to be an effective treatment more than a decade ago,^15^ current societal guidelines still recommend surgical decortication as the primary management strategy.^1,15,16,17^ More recently, a consensus statement was published in which IPFT is recommended as the initial management for patients with a complicated parapneumonic effusion, but surgery is recommended for patients with an empyema, depending on local expertise and minimally invasive service availability.^18^

High-quality, adequately powered prospective randomized clinical trials are needed to definitively compare these 2 management strategies and guide clinical decision-making. This pilot study of CPI management compared chest tube drainage of the pleural cavity using IPFT and surgical decortication with the intent to inform on the study algorithm and end point performance in anticipation of a multicenter randomized clinical trial.

## Methods

### Study Design

This pilot parallel randomized clinical trial was conducted at a single center in Seattle, Washington, between March 1, 2019, and December 31, 2021, with follow-up for 90 days. Ethical and regulatory approval for the study was obtained from the Providence St Joseph Health Institutional Review Board. All participants provided written informed consent. We followed the Consolidated Standards of Reporting Trials (CONSORT) reporting guideline. All patients received standard of care management except for randomization to treatment group and chest tube removal criteria. The trial protocol is available in Supplement 1.

### Patient Identification and Eligibility

Patients admitted to or consulting with the Department of Thoracic Surgery and Interventional Pulmonology were screened for eligibility daily. Patients were eligible if they were 18 years or older, had a clinical pleural infection (fever and/or leukocytosis, elevated procalcitonin level, and/or elevated C-reactive protein level) and positive results of pleural fluid analysis (macroscopically purulent, positive Gram stain or culture, lactate dehydrogenase level >1000 U/L [to convert to microkatals per liter, multiply by 0.0167], or glucose level <40 mg/dL [to convert to millimoles per liter, multiply by 0.0555]). Pleural fluid sampling and timing was performed as per standard clinical care. Exclusion criteria are provided in eTable 1 in Supplement 2. Data on self-reported race and ethnicity (Asian, Black, Hispanic, and White) were collected to describe the population characteristics.

### Trial Pathway and Randomization

**Figure 1.**
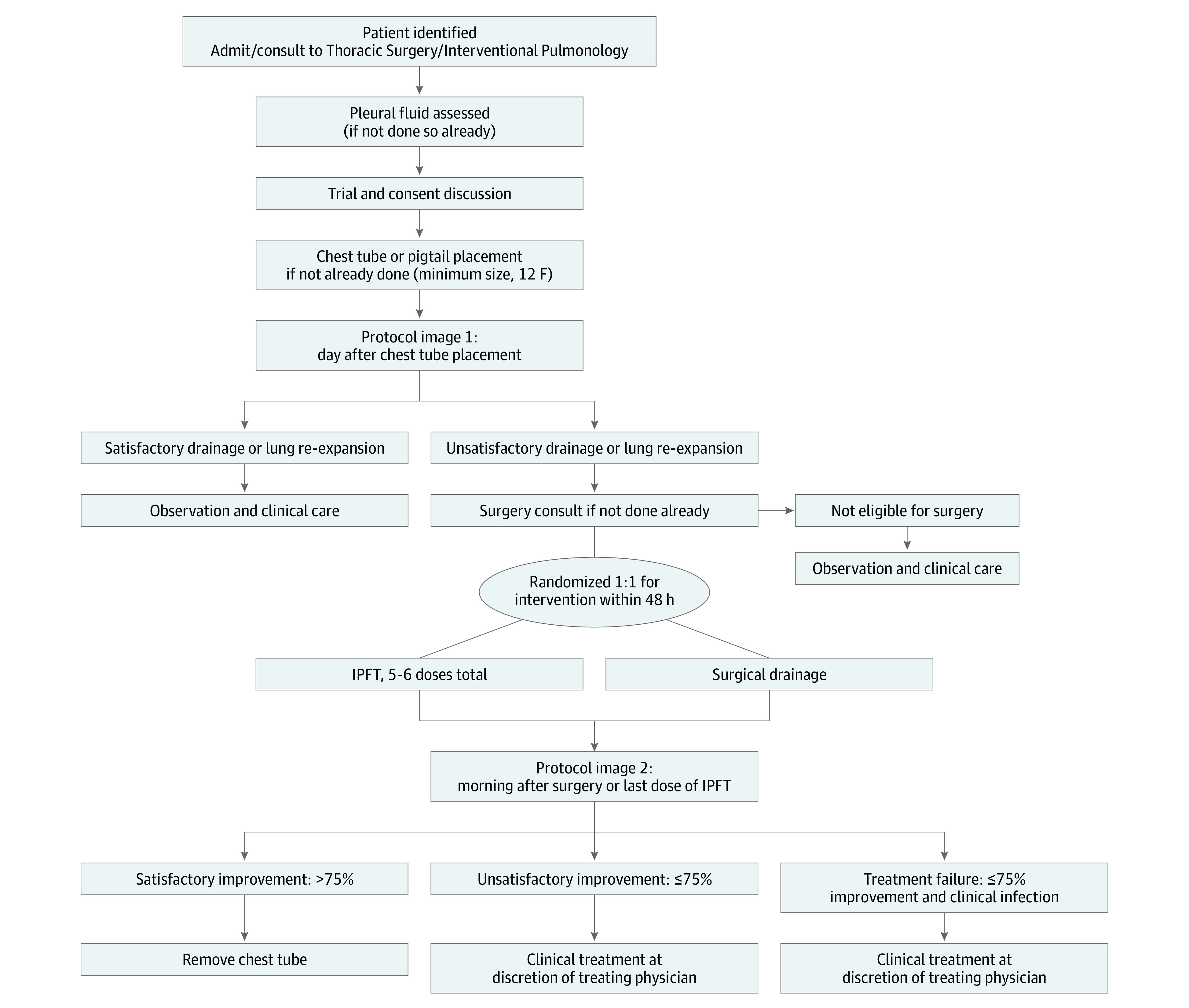
Randomized Clinical Trial Algorithm IPFT indicates intrapleural fibrinolytic therapy.

Once eligible patients were identified, managing physicians discussed the trial with them, and written informed consent was obtained (Figure 1). All patients underwent tube thoracostomy for pleural cavity drainage, if not already performed. The minimum chest tube size was initially set to 14F but was later changed to 12F as discussed hereinafter.

Effectiveness of drainage and/or lung re-expansion was determined on protocol image 1, defined as imaging (chest radiography or chest computed tomography) the day following chest tube placement. Patients with complete pleural fluid drainage and adequate lung re-expansion were followed up with usual clinical care without randomization and were observed until discharge. Patients with incomplete drainage and/or inadequate lung re-expansion as assessed by the attending physician underwent a thoracic surgery consultation to determine surgical candidacy. Those patients deemed ineligible for surgical intervention received guideline-appropriate nonsurgical care and were observed until discharge.

Those eligible for surgical management underwent randomization with a 1:1 allocation using the sealed envelope technique, generated by the research coordinator, to either IPFT or surgery. The primary intervention was required to be initiated within 48 hours. Those patients randomized to the IPFT group received 5 to 6 doses of IPFT with 10 mg of tissue plasminogen activator and 5 mg of deoxyribonuclease delivered twice a day through the chest tube, which was then clamped, allowing the IPFT to remain in the pleural cavity for 1 hour. Patients randomized to the surgical group underwent surgical decortication with either an open or a video-assisted thoracoscopic surgery (VATS) approach at the discretion of the surgeon.

The morning following intervention completion (last dose of IPFT or surgery), protocol image 2 was obtained to categorize drainage as satisfactory improvement of the pleural fluid collection (>75% improvement in opacity on imaging), unsatisfactory improvement (≤75% improvement without clinical evidence of infection), or treatment failure (≤75% improvement and evidence of ongoing infection, defined below). In patients with satisfactory improvement without ongoing signs and symptoms of infection, chest tubes were removed once fluid was serous and output was less than 200 mL in 24 hours. In patients with unsatisfactory improvement without ongoing signs and symptoms of infection, and in those with treatment failure, further clinical management was at the discretion of the managing physician.

### Objectives and Outcomes

The primary objective was to determine the feasibility of the proposed study algorithm comparing IPFT and surgical intervention as measured by percentage of enrollment to completion of the study algorithm and percentage of multidisciplinary participation in adherence to the algorithm. There were multiple secondary objectives evaluated in an effort to maximize successful recruitment, assist with multicenter study design, and obtain preliminary information for sample size estimates. We evaluated patient identification strategies (number of and reason for inadequate screening of eligible patients and failure of screening to enrollment) and collected accrual times (time to accrual of 20 patients or number of patients accrued in 1 year, whichever occurred first). Preliminary clinical data on the proposed chest tube management algorithm were also collected, including number of chest tube and hospital days. Supplementary clinical end points (radiographic improvement, treatment failures, additional procedures, complications, and 30- and 90-day mortality) were collected to inform on the efficacy and safety of the algorithm.

### Definitions

Comorbidities were classified according to the Charlson Comorbidity Index.^19^ A CPI was classified as either complicated parapneumonic effusion (loculated pleural effusion with positive biochemical pleural fluid profile) or empyema (purulent pleural fluid), using the definition outlined in the American College of Chest Physicians consensus statement.^17^ Complications were categorized according to the Ottawa classification of thoracic morbidity and mortality.^20^ Complications were only recorded for the initial admission; treatment failures and crossovers were not reported as complications. In patients with multiple complications, the highest grade of complication was recorded. Duration of chest tube use was defined as total number of days from the first day of the intervention (day of first dose of IPFT or day of surgery) to the day of the last chest tube removal; for observation patients, it was defined from first chest tube insertion to removal of the last chest tube. Hospital length of stay was defined as total number of days from the first day of the intervention (day of first dose of IPFT or day of surgery) to the date of discharge; for observation patients, it was defined from the date of first chest tube insertion to the date of discharge.

Treatment failure was defined by evidence of ongoing infection, including fever and/or leukocytosis and a persistent pleural collection still present at least 48 hours following the initial intervention. Crossover treatment, independent of treatment failure, was defined by receiving any dose of IPFT following surgery or surgery following any dose of IPFT. The RAPID (Renal, Age, Purulence, Infection Source, and Dietary Factors) score for pleural infection was calculated as a validated factor associated with mortality.^21^

### Statistical Analysis

As this was a pilot study testing feasibility, we aimed to enroll a total of 30 patients, with the goal of randomizing 20 patients (10 in each group) and consideration for patients not randomized falling into the observation group. Groups were compared using an intention-to-treat analysis.

Continuous data were reported as medians with IQRs. Categorical and count data were presented as frequencies and percentages. Characteristics between the study groups were compared using χ^2^ tests for categorical variables and the Mann-Whitney test for continuous variables. Groups were compared with the use of the Yates correction and Fisher exact test, where appropriate. All tests were 2 sided, and statistical significance was set at *P* < .05. Statistical analyses were performed using RStudio, version 2022.02.3 (R Project for Statistical Computing).

## Results

### Algorithm Feasibility

A total of 26 patients were enrolled, with 10 in each treatment group and 6 in the observation group. There was 100% enrollment of patients in each treatment group to study completion. Although there were no health care professional protocol deviations in consented patients, 2 thoracic surgeons voiced a preference for surgery in 3 patients (3 of 48 exclusions [6%]) screened for inclusion. These patients were excluded prior to consent.

Two amendments were made to the protocol during the trial period. The first was to drop the minimum size of the initial chest tube from 14F to 12F, as outside hospitals were referring patients with 12F chest tubes. The second was a change to the wording for protocol image 1 to “next day” instead of “within 24 to 48 hours,” as specific time restraints were restrictive for patient care.

### Patient Identification and Accrual

**Figure 2.**
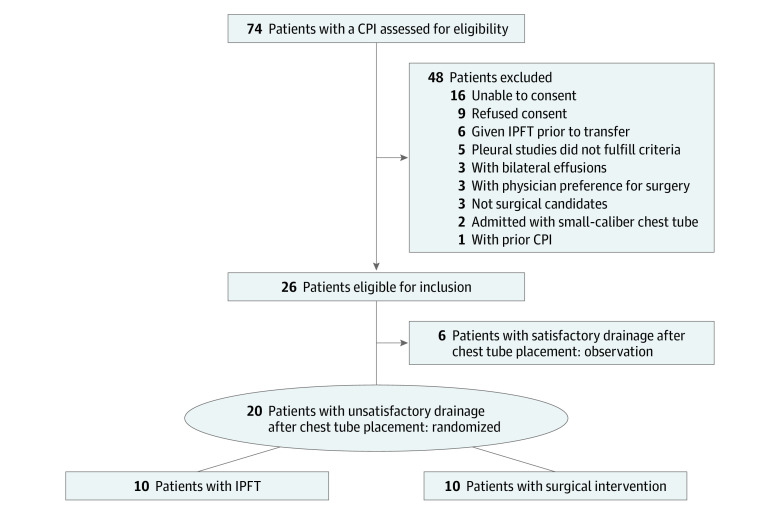
Flow Diagram of Trial Profile CPI indicates complicated pleural infection; IPFT, intrapleural fibrinolytic therapy.

Seventy-four patients with a CPI were assessed for eligibility over the study period, and 48 (65%) were excluded (Figure 2). There was no screening to enrollment failure, and all eligible patients admitted to or consulting with our department were screened. It took 32 months to enroll 26 patients (9 in 2019, 8 in 2020, and 9 in 2021). Of the 26 included patients, 16 (62%) were transferred from an outside hospital.

### Clinical Outcomes of Randomized Patients

Overall, the 20 randomized patients had a median age of 57 (IQR, 46-65) years; 15 (75%) were men and 5 (25%) were women (Table 1). One patient (5%) was Asian, 1 (5%) was Black, 1 (5%) was Hispanic, and 17 (85%) were White. All CPIs were community-acquired infections (20 [100%]), most patients had complicated pleural infections (12 [60%]) vs empyemas (8 [40%]), and the median RAPID score was 2 (IQR, 1-3). Characteristics were similar when stratified across IPFT and surgical groups. The microbiology identified from pleural infections with a positive pleural fluid culture for the total population is shown in eTable 2 in Supplement 2.

**Table 1.** Summary Statistics of Baseline Measures Overall and Stratified by Treatment Group

Demographic characteristic	Study group^a^		
	Overall (n = 20)	IPFT (n = 10)	Surgery (n = 10)
Age, median (IQR) y	57 (46-65)	55 (41-62)	57 (50-70)
Sex			
Men	15 (75)	7 (70)	8 (80)
Women	5 (25)	3 (30)	2 (20)
Body mass index, median (IQR)^b^	26 (22-30)	24 (22-33)	27 (24-29)
Race and ethnicity			
Asian	1 (5)	1 (10)	0
Black	1 (5)	1 (10)	0
Hispanic	1 (5)	1 (10)	0
White	17 (85)	7 (70)	10 (100)
Relevant comorbidities			
Chronic obstructive pulmonary disease	2 (10)	1 (10)	1 (10)
Diabetes	2 (10)	1 (10)	1 (10)
Malnutrition	1 (5)	0	1 (10)
Immunosuppression	1 (5)	1 (10)	0
Malignant neoplasm	1 (5)	1 (10)	0
Intravenous drug use	2 (10)	0	2 (20)
HIV and/or AIDS	1 (5)	1 (10)	0
Charlson Comorbidity Index			
0	4 (20)	2 (20)	2 (20)
1-2	5 (25)	1 (10)	4 (40)
3-4	8 (40)	5 (50)	3 (30)
≥5	3 (15)	2 (20)	1 (10)
Community-acquired infection	20 (100)	10 (100)	10 (100)
CPI phase			
Complicated	12 (60)	6 (60)	6 (60)
Empyema	8 (40)	4 (40)	4 (40)
Positive fluid Gram stain	7 (35)	4 (40)	3 (30)
Positive fluid culture	11 (55)	4 (40)	7 (70)
RAPID score, median (IQR)	2 (1-3)	2 (1-4)	2 (1-3)
Evidence of loculation on protocol image 1	12 (60)	6 (60)	6 (60)

Abbreviations: CPI, complicated pleural infection; IPFT, intrapleural fibrinolytic therapy; RAPID, Renal, Age, Purulence, Infection Source, and Dietary Factors.

^a^ Unless otherwise indicated, data are expressed as No. (%) of patients. Percentages have been rounded and may not total 100.

^b^ Calculated as weight in kilograms divided by height in meters squared.

In those patients randomized to IPFT, 6 (60%) had 6 doses, 2 (20%) had 5 doses, and 2 (20%) had 4 doses. One patient who received 4 doses refused further IPFT due to chest pain and requested surgery. This patient underwent crossover treatment and received a VATS decortication. The other patient who received 4 doses had a hemothorax, IPFT was stopped, and the hemothorax was monitored. The patient’s CPI ultimately resolved with no further treatment. In those patients randomized to surgery, all patients received a VATS decortication.

On protocol image 2 following completion of treatment, 5 patients (50%) in the IPFT group and 3 (30%) in the surgery group had greater than 75% opacification improvement (Table 2). Only 1 patient, who was in the IPFT group and completed 6 doses of IPFT, had treatment failure. This patient was considered a surgical candidate at study enrollment but due to a complicated clinical course, this patient was deemed ineligible for surgical decortication at treatment failure and underwent empyema tube placement. Two patients underwent crossover treatment, both were in the IPFT group, and neither experienced treatment failure. The first patient was discussed above, and the second patient had an empyema with extensive loculations and marginal pulmonary re-expansion following IPFT, who subsequently underwent open decortication. Intraprocedure and postprocedure complication rates were similar across the IPFT and surgery groups. Details of major complications are shown in Table 3. There were no reoperations and no in-hospital deaths.

**Table 2.** Clinical and Outcome Details Overall and Stratified by Treatment Group

Outcome	Treatment group^a^			*P* value, IPFT vs surgery
	Overall (n = 20)	IPFT (n = 10)	Surgery (n = 10)	
Treatment outcomes				
Radiographic improvement, %				
<50	6 (30)	3 (30)	3 (30)	.80
50-75	6 (30)	2 (20)	4 (40)	
>75	8 (40)	5 (50)	3 (30)	
Treatment failure	1 (5)	1 (10)	0	>.99
Crossover treatment	2 (10)	2 (20)	0	.46
Crossovers that were treatment failures	0	0	0	>.99
Additional treatment	2 (10)	2 (20)	0	
Empyema tube	1 (5)	1 (10)	0	.46
Surgical decortication	1 (5)	1 (10)	0	
Complications				
Intraprocedure	2 (20)	0	2 (20)	
Diaphragmatic injury	1 (5)	0	1 (10)	.46
Hypotension and hypoxia	1 (5)	0	1 (10)	
Postprocedure^b^	15 (75)	9 (90)	6 (60)	.30
Minor	8 (40)	4 (40)	4 (40)	
Grade I	2 (10)	2 (20)	0	.65
Grade II	6 (30)	2 (20)	4 (40)	
Major	7 (35)	5 (50)	2 (20)	
Grade IIIa	3 (15)	2 (20)	1 (10)	.35
Grade IVa	4 (20)	3 (30)	1 (10)	
Mortality				
Grade V	0	0	0	>.99
Admission details				
Duration of chest tube use, median (IQR), d	4 (4-6)	5 (4-8)	4 (3-5)	.21
Discharged with chest tube in situ	3 (15)	2 (20)	1 (10)	>.99
ICU length of stay, median (IQR), d	0 (0-2)	0 (0-3)	0 (0-1)	.28
Hospital length of stay, median (IQR), d	6 (4-9)	11 (4-18)	5 (4-6)	.08
Discharge location				
Home	16 (80)	8 (80)	8 (80)	.59
Skilled nursing facility	2 (10)	2 (20)	0	
Against medical advice	2 (10)	0	2 (20)	
30-Day readmission	0	0	0	>.99
30-Day mortality	0	0	0	>.99
90-Day mortality	0	0	0	>.99

Abbreviations: ICU, intensive care unit; IPFT, intrapleural fibrinolytic therapy.

^a^ Unless otherwise indicated, data are expressed as No. (%) of patients. Percentages have been rounded and may not total 100.

^b^ Grade I indicates any complication without need for pharmacological treatment or other intervention; grade II, any complication that requires pharmacological treatment or minor intervention only; grade III, any complication that requires surgical, radiological, or endoscopic intervention or multitherapy; grade IIIa, intervention does not require general anesthesia; grade IV, any complication requiring intensive care unit management and life support; grade IVa, single organ dysfunction; grade V, any complication leading to the death of the patient.

**Table 3.** Major Postprocedure Complication Details, Stratified by Treatment Group

Major complication^a^	Study group	
	IPFT (n = 10)	Surgery (n = 10)
Grade IIIa		
No. (%) of patients	2 (20)	1 (10)
Complication	Acute kidney failure and intra-abdominal chest tube placement	Hematemesis with aspiration
Grade IVa		
No. (%) of patients	3 (30)	1 (10)
Complication	Persistent air leak with mucus plugging; hypotension postoperatively (crossover); hypoxemic respiratory failure	Severe hypotension and/or bradycardia postoperatively

Abbreviation: IPFT, intrapleural fibrinolytic therapy.

^a^ Grade IIIa indicates intervention does not require general anesthesia; grade IVa, single organ dysfunction.

The median duration of chest tube use was comparable in the IPFT (5 [IQR, 4-8] days) and surgery (4 [IQR, 3-5] days) groups (*P* = .21) (Table 2). The criteria set for chest tube removal was adequate, as no additional procedures were required once the chest tube was removed in all patients. Three patients (15%) were discharged with a chest tube in situ, of whom 2 were in the IPFT group. Median hospital length of stay tended to be longer in the IPFT group (11 [IQR, 4-18] days) compared with the surgery group (5 [IQR, 4-6] days), although not significantly (*P* = .08). There were no 30-day hospital readmissions or 30- or 90-day deaths.

### Observation Patients

There were 6 patients in the observation group. Their median RAPID score was 3 (IQR, 2-4), and 4 (67%) had a complicated pleural infection vs 2 (33%) who had an empyema. During observation, 2 patients (33%) were given additional treatment for radiographic persistent pleural fluid collections; neither had a fever or leukocytosis at the time of additional treatment. Both patients received IPFT; one patient had a single dose and the other had 3 doses. Protocol image 1 for both of these patients was chest radiography.

Only 1 patient (17%) had a complication (grade II): a dental abscess. The median duration of chest tube use was 4 (IQR, 3-6) days, and the median hospital length of stay was 10 (IQR, 7-13) days. One patient (17%) who required additional treatment was readmitted within 30 days with a persistent parapneumonic effusion, a new contralateral pleural effusion, and acute hypoxic respiratory failure. The patient underwent chest tube placement and 3 doses of IPFT. There were no 30- or 90-day deaths in the observation group.

## Discussion

In this pilot randomized clinical trial, we identified the study algorithm comparing IPFT with surgical management of a CPI to be feasible with 100% patient enrollment to study completion and excellent multidisciplinary protocol adherence following randomization. Patient identification and accrual was adequate, with all eligible patients screened, no screening to enrollment failures, and approximately 9 patients enrolled annually. Clinical characteristics and outcomes were similar to those in previous reports, confirming safety and efficacy of the algorithm. We also conclude that our standardized chest tube removal criteria were effective, as no additional procedures were required in randomized patients following chest tube removal. We believe these findings provide evidence that the proposed algorithm can move forward to a large, multicenter randomized clinical trial.

Previous studies^2,9,10,13,22,23,24,25^ have compared surgery with single-agent IPFT or with nonoperative management. There are, however, significant limitations to these studies, including underpowered statistics and inadequate or heterogenous categorization of the nonoperative management group. A previous publication,^11^ although retrospective, identified surgical intervention to have advantages over IPFT. Additionally, surgical management of CPI was identified as declining as IPFT becomes more prevalent, and there is large variation in IPFT dosing regimens.^11,26,27,28,29^ Current guidelines, both medical and surgical, lack comparative trials on which to base recommendations.^1,16,17^

Supporting the primary outcome, we identified the study algorithm to be feasible, safe, and efficacious. As with any randomized trial, equipoise in physician screening of patients and in discussion of the trial with patients is critical to study the algorithm feasibility and implementation. With the proposed algorithm, there were 3 instances of the physician choosing surgery instead of randomization, and these patients were excluded prior to enrollment. This may be prevented in the future by including sites where physicians commit to the algorithm. At the study summation meeting of investigators, all participants voiced support for the study algorithm and to proceed with a multicenter trial.

A potential challenge to accrual, which should not affect future trials, was the emergence of the COVID-19 pandemic. Study enrollment was paused briefly in 2020, and while this may have prolonged the overall duration of patient enrollment, enrollment was similar in 2020 and 2021 compared with 2019. Patient clinical follow-up was likely affected, as we noticed patients were not routinely available.

This study was not powered to identify differences in clinical outcomes; therefore, the small population size warrants some hesitation in interpretation. However, with that caveat, our outcomes appear relatively similar to the previously published retrospective data.^11^ We believe a strength of our trial is the low rate of nonsurgical candidates during the screening process. During recruitment, only 3 patients were initially considered nonsurgical. These 3 patients were excluded for end-stage hepatic and kidney disease, which would also be common exclusion criteria for many other prospective trials. Additionally, our data contrasts potential notions that many high-risk, sick individuals are being shunted to IPFT treatment. We also captured the outcomes of individuals in the observation group, a population unavailable in the prior retrospective review.^11^ Within this small subset, we identified 2 patients who underwent additional therapy (IPFT), and 1 was readmitted within 30 days. These patients may have benefited from protocol image 1 being a computed tomographic scan instead of chest radiographic image, with the possibility of undergoing randomization and further treatment up front.

This pilot trial is notable as it is one of the first to assess the feasibility, safety, and efficacy of a study algorithm comparing IPFT with surgical decortication for the management of a CPI. Ongoing trials across the globe^30,31^ and the MIST-3^32^ assessing the feasibility of randomizing patients with CPI to early IPFT or VATS will add to the evidence base surrounding this clinical dilemma, but they remain exploratory in their design.^33^ We believe there remains a need and value in performing an appropriately powered multicenter randomized clinical trial within the US to better address care for this common and complex problem.

### Limitations

This study has some limitations. We identified chest tube duration as a clinically useful primary outcome that is both easy to quantify and standardize; therefore, we intend to use chest tube duration as the primary outcome in the future multicenter trial. Due to the small sample size and skewed mean values in this pilot study, the power calculation will have to be based on cumulative historical data. Another limitation of this study is the use of the Ottawa classification of thoracic morbidity and mortality for categorizing complications. This classification was designed for postoperative thoracic surgery complications, and it is also based on the severity of the complication being proportional to the effort to treat it. We would consider a hemothorax due to IPFT to be a significant complication; however, since IPFT was stopped and there was no further treatment, this complication is categorized as minor. We used this classification in an attempt to standardize categorization of complications for comparison purposes.

## Conclusions

In this pilot randomized clinical trial, we identified an algorithm comparing tissue plasminogen activator plus deoxyribonuclease therapy with surgical decortication to be feasible with 100% enrollment to completion, with excellent multidisciplinary participation and adherence. These findings show that the studied algorithm is safe and efficacious for patients with complicated pleural infections, with a strong feasibility for appropriate enrollment within a reasonable time frame for a multicenter study.

## Notes

Supplement 1.
Trial Protocol

Supplement 2. **eTable 1.** Exclusion Criteria **eTable 2.** Microbiology Identified in Pleural Infections With a Positive Pleural Fluid Culture for the Total Population (N = 26)

Supplement 3.
Data Sharing Statement
