# Supplementary material for: Effect of Intrapleural Fibrinolytic Therapy vs Surgery for Complicated Pleural Infections: A Randomized Clinical Trial
Source: JAMA Netw Open. 2023 Apr 12;6(4):e237799. doi: 10.1001/jamanetworkopen.2023.7799 (PMC10098968; doi:10.1001/jamanetworkopen.2023.7799)
Supplement: Supplement 1. — Trial Protocol [file jamanetwopen-e237799-s001.pdf]

PROTOCOL TITLE:

Management of pleural space infections: A randomized pilot study

PRINCIPAL INVESTIGATOR:

Jed Gorden, MD
Swedish Cancer Institute and Medical Center- Interventional Pulmonology
1101 Madison Street, Suite 900
Seattle, WA 98104
(206) 215-6800
Jed.gorden@swedish.org

Eric Vallières, MD, FRCSC
Swedish Cancer Institute and Medical Center- Thoracic Surgery
1101 Madison Street, Suite 900
Seattle, WA 98104
(206) 215-6800
Eric.vallieres@swedish.org

Sub Investigators:

Christopher Gilbert, DO, MS
Swedish Cancer Institute and Medical Center- Interventional Pulmonology
1101 Madison Street, Suite 900
Seattle, WA 98104
(206) 215-6800
Christopher.gilbert@swedish.org

Candice Wilshire, MD
Swedish Cancer Institute and Medical Center- Interventional Pulmonology
1101 Madison Street, Suite 900
Seattle, WA 98104
(206) 215-6800
Candice.wilshire@swedish.org

Brian Louie, MD
Swedish Cancer Institute- Thoracic Surgery
Director of Research and Education
Co-director, MIS Thoracic Surgery Program
1101 Madison Street
Suite 900
(206) 215-6800
brian.louie@swedish.org

Ralph Aye, MD, FACS
Swedish Cancer Institute – Thoracic Surgery
Program Leader, Thoracic Oncology
1101 Madison Street, Suite 900
Seattle, WA 98104
(206) 215-6800
ralph.aye@swedish.org

Alex Farivar, MD
Swedish Cancer Institute – Thoracic Surgery
1101 Madison Street, Suite 900
Seattle, WA 98104
(206) 215-6800
alex.farivar@swedish.org

Adam Bograd, MD
Swedish Cancer Institute – Thoracic Surgery
1101 Madison Street, Suite 900
Seattle, WA 98104
(206) 215-6800
adam.bograd@swedish.org

VERSION NUMBER/DATE:

Version Number: 4.0

Date: 5/2/19

REVISION HISTORY

Revision #	Version Date	Summary of Changes	Consent Change?
3	5/2/19	• Minimum chest tube size was changed to 12 F as it is the practice of many outside hospitals to put 12F chest tubes in<br>• The wording of the timing for imaging 1 has been amended to make it clear that the image needs to be obtained the next day, not necessarily a full 24 hours after placement. This was changed to be in line with usual clinical care and to not disrupt the flow of patient care.	Updates to consent to match this
4	7/12/2019	• Change 30-day and 90-day follow-up to	Updates to

		telephonic follow-up instead of in-person follow-up	consent to match this

Table of Contents

1.0 Study Summary 5
2.0 Objectives* 8
3.0 Background* 8
4.0 Study Endpoints* 8
5.0 Study Intervention/Investigational Agent 9
6.0 Procedures Involved* 10
7.0 Data and Specimen Banking* 14
8.0 Sharing of Results with Subjects* 15
9.0 Study Timelines* 15
10.0 Inclusion and Exclusion Criteria* 15
11.0 Vulnerable Populations* 16
12.0 Local Number of Subjects 16
13.0 Recruitment Methods 16
14.0 Withdrawal of Subjects* 17
15.0 Risks to Subjects* 17
16.0 Potential Benefits to Subjects* 18
17.0 Data Management* and Confidentiality 18
18.0 Provisions to Monitor the Data to Ensure the Safety of Subjects* 18
19.0 Provisions to Protect the Privacy Interests of Subjects 20
20.0 Compensation for Research-Related Injury 20
21.0 Economic Burden to Subjects 20
22.0 Consent Process 20
23.0 Process to Document Consent in Writing 21
24.0 Setting 21
25.0 Resources Available 21
26.0 Multi-Site Research* 22

1.0 Study Summary

**Study Title**	**Management of pleural space infections: A randomized pilot study**
**Study Design**	Single center, Randomized trial
**Primary Objective**	• Test the feasibility of the proposed study algorithm to compare Intrapleural fibrinolytic therapy (IPFT) to surgical intervention as measured by percent enrollment to completion of study algorithm and multidisciplinary participation in adherence to the algorithm.
**Secondary Objective(s)**	• Optimize patient identification strategies and gauge accrual timeline to assist with multicenter study planning and budget needs in terms of number of personnel needed to perform the study.<br><br>• Collect preliminary clinical data on the proposed chest tube management algorithm, including chest tube days (to be used for power calculations in larger trial), as well as efficacy and safety of chest tube management algorithm.
**Research Intervention(s)/ Investigational Agent(s)**	**IPFT**: The IPFT group will receive a total of 5-6 doses of alteplase and DNase delivered through a chest tube or small bore catheter into the pleural space. The doses will be given twice a day. If the first IPFT dose is given in the evening on the first day, the patient will only receive a total of 5 doses.<br><br>or<br><br>**Surgery**: The surgical group will receive either open surgery or a video-assisted thoracoscopic (VATS) approach at the discretion of the surgeon.
**IND/IDE #**	
**Study Population**	<u>**Inclusion Criteria:**</u><br><br>• Age >18 years<br>• Clinical presentation compatible with pleural infection (fever or leukocytosis, elevated procalcitonin, elevated C-reactive protein (CRP))<br>• Pleural fluid requiring drainage that is either:

- Macroscopically purulent **or**
- Positive on culture for bacterial infection **or**
- Positive for bacteria on gram stain **or**
- Lactate dehydrogenase (LDH) > 1000 IU/L **or**
- Glucose <40 mg/dL

<u>Exclusion Criteria:</u>

- Age <18 years
- Unable to give consent (No surrogate consent of legally authorized representatives allowed for this study)
- Not proficient in English
- History of prior ipsilateral complicated pleural space infection
- Has known sensitivity to DNase or alteplase
- History of intracranial hemorrhage or acute intracranial hemorrhage
- History of stroke, hemorrhage, or trauma within the last 3 months
- Has had prior surgery on the side of the pleural infection
- Patients who are pregnant or lactating
- Expected survival less than 6 months from a different pathology to this pleural infection based on clinical judgment
- Has a tunneled pleural catheter in place
- Patients on anticoagulation that cannot be interrupted for surgical intervention
- Patients with known or suspected malignant pleural effusion
- Patients with renal failure (Creatinine clearance <30)
- Prior history of or concern for chylothorax or pseudochylothorax
- Vulnerable populations: prisoners
- Hemothorax
- Kaiser-Permanente Patients (Due to contracting barriers)
- Evidence of clinically significant bilateral effusions at time of evaluation

	• Intrapleural fibrinolytic therapy given prior to study screening
**Sample Size**	30
**Study Duration for individual participants**	From consent during hospital admission to 1-year follow up post discharge
**Study Specific Abbreviations/ Definitions**	IPFT=Intrapleural Fibrinolytic Therapy<br>VATS= Video Assisted Thoracoscopic Surgery

2.0 Objectives*

Primary Objective:

- Test the feasibility of the proposed study algorithm to compare IPFT to surgical intervention as measured by percent enrollment to completion of study algorithm and multidisciplinary participation in adherence to the algorithm.

Secondary Objectives:

- Optimize patient identification strategies and gauge accrual timelines to assist with multicenter study planning and budget needs in terms of number of personnel needed to perform the study.

- Collect preliminary clinical data on the proposed chest tube management algorithm, including chest tube days (to be used for power calculations in larger trial), as well as efficacy and safety of chest tube management algorithm.

3.0 Background*

Complex pleural space infections are a common and morbid clinical condition affecting approximately 60,000 patients annually in the United States with a nearly 15% mortality rate.[1,2] Effective treatment requires both antibiotic therapy and drainage of the pleural space. Since IPFT therapy has previously been ineffective in clinical trials[3], surgery is the standard of care for complex pleural space infection drainage and is currently recommended by the American College of Chest Physicians (ACCP),the British Thoracic Society (BTS), and American Association of Thoracic Surgeons (AATS).[4–6] However, more recent data has shown effective drainage of pleural space infections using dual agent intrapleural fibrinolytic therapy (IPFT) administration at the bedside via a small caliber chest tube using Alteplase and DNase alpha.[7] There now exists two very different, and competing drainage strategies (IPFT and surgical drainage) that need to be considered in clinical decision-making.

Currently, there is no high-quality evidence comparing the clinical outcomes and cost effectiveness of surgical drainage combined with antibiotics versus dual-agent IPFT catheter drainage of pleural space infections with concomitant antibiotic therapy**.** The absence of comparative data is a challenge for surgical and medical services in clinical decision-making for this common and morbid condition.

This is a pilot study comparing surgical drainage of the pleural space in complex pleural effusions to bedside chest tube drainage using dual agent IPFT with the intent to inform on study algorithm and endpoint performance in anticipation of a multi-institutional randomized clinical trial.

4.0 Study Endpoints*

<u>Outcome Measures for Objectives Collected in Pilot Study:</u>

1. **Feasibility of algorithm**
   - Percent of subjects enrolled to study completion
   - Percent of subjects randomized but did not complete study, and reason
   - Percent of health care professional protocol deviation, specialty and reason
2. **Subject identification and accrual**
   - Screened to enrollment failures
   - Percent of eligible patients not screened and randomized, and reason
   - Time to accrual of 20 patients or number of patients accrued in one year, whichever occurs first
3. **Clinical outcomes**
   - <u>Clinical outcomes primary endpoint for future study:</u>
     - Chest tube duration measured from initiation of intervention (IPFT or surgical debridement/decortication) to removal. Study algorithm governs chest tube removal parameters.
   - <u>Clinical outcomes secondary endpoints:</u>
     - Radiographic improvement (described in Section 6.0).
     - Treatment failures needing treatment crossover (IPFT to surgery, surgery to IPFT) with treatment failures defined as ongoing signs of infection with residual pleural fluid collection evident on imaging.
     - Additional procedures performed within 30 days
     - Procedure-related complications (documented using the Common Terminology Criteria for Adverse Events (CTCAE))
     - 30-day,90 day, and 1-year outcomes including quality of life as measured by the RAND 36-Item Short Form Survey Instrument (SF-36) (Appendix 3) and Work status Questionnaire (Appendix 4-7).
     - Mortality
     - Cost comparison

5.0 Study Intervention/Investigational Agent

Patients will be randomized into either initial IPFT or surgery:

a. **IPFT**: The IPFT group will receive a total of 5-6 doses of alteplase 10mg and 5 DNase mg BID x 3 days delivered through a chest tube or small bore catheter into the pleural space. The doses will be given twice a day. If the first IPFT dose is given in the evening on the first day, they will only receive a total of 5 doses of the dual-agent IPFT (alteplase and DNase).

b. **Surgery**: The surgical arm will have either open surgery or a VATS approach at the discretion of the surgeon.

IPFT (alteplase and DNase) is not an investigational agent. It is used in standard of care practice for the treatment of complex pleural space infections. We seek to compare dual-agent IPFT (alteplase and DNase) to surgery in this study. As such, the IPFT agents will be ordered from pharmacy through the electronic medical record (EMR) as in normal practice and there is not a study drug.

6.0 Procedures Involved*

This is a pilot study comparing surgical drainage of the pleural space in complex pleural effusions to bedside chest tube drainage using dual agent IPFT with the intent to inform on study algorithm and endpoint performance in anticipation of a multi-institutional randomized clinical trial. The study is a randomized clinical trial design performed at a single institution, Swedish Medical Center First Hill (see attached Appendix 1 flowsheet of algorithm).

Study Algorithm:

Patient identification: All inpatients admitted to Swedish Medical Center First Hill campus admitted under the care of the Thoracic Surgery/Interventional Pulmonology service or seen in consultation by the Thoracic Surgery/Interventional Pulmonology service are eligible to be screened for participation. Members of the Kaiser-Permanente health care plan will be excluded due to contracting barriers. Overnight and daytime teams will be contacted daily to identify patients appropriate for screening.

Clinical baseline metrics: All patients will have standard of care laboratories collected including: complete blood count (CBC), procalcitonin, complete metabolic panel (CMP), C-reactive protein (CRP), liver function tests (LFTs), and coagulation studies. Vital signs including heart rate, blood pressure, and temperature will be recorded, as per standard of care. If not already initiated, antibiotics targeted to cultures or hospital infectious disease consult recommendation will be started.

Pleural sampling: Pleural fluid may be sampled by the responsible clinical service in the course of clinical care for patients with suspected pulmonary infection or by consulting service. Some patients will have a thoracentesis or tube drainage, and others will have no intervention at the time of referral for study eligibility review. Enrollment reflects real world clinical care and patients will be considered for enrollment regardless of sampling techniques or timing prior to evaluation.

Screening and consent: Patients who meet all inclusion criteria and have no exclusion criteria are eligible for screening. A designated member of the clinical team will approach participants who fulfil the criteria for inclusion into the trial.

A patient information sheet (consent form describing the study) will then be given to the participant; the doctor/research team will then discuss the study aims, design and

potential risks involved, as well as alternatives to participation. If participants are willing to take part in the study they will need to give written consent. Consent will be obtained at this time.

Pleural fluid drainage: All patients in the study must have tube thoracostomy for drainage of the pleural space. This may have been done by the clinically responsible team or by the study team. Physicians may select chest tubes of any type, but the minimum size is 12 French. For patency, chest tubes 12-20 French should be kept at negative 20 suction and flushed daily. Large bore chest tubes (>20French) may be kept at negative 20 suction at the discretion of the physician managing the chest tube and do not require daily flushing. Supportive care will follow Institutional Guidelines.

Protocol Image #1: Once the chest tube is placed, imaging is obtained the next day to assess the fluid drainage. This does not include the immediate post chest tube placement image obtained. The choice of image, either Chest X-ray or CT Chest, is up to the discretion of the treating physician. Based on the imaging, patients will be separated into 2 groups:

A: Complete drainage/re-expansion of the lung: If there is complete drainage of the pleural fluid collection and lung re-expansion based on image #1, patients will follow usual clinical care without randomization and be observed until discharge. Patients will remain in the study and their data will be collected.

B: Incomplete drainage/incomplete lung re-expansion: For those patients that have incomplete drainage of the pleural fluid collection on image #1 and/or the lung does not re-expand.

Surgical Consultation: A thoracic surgery consultation will be obtained on all patients in group b, incomplete drainage and/or the lung does not re-expand, to determine surgical candidacy. Those patients that the surgical team deem unsafe for surgery will receive clinically appropriate guideline centered, non-surgical, care. These patients will not be randomized but they will remain in the study and their data will be collected. Criteria deeming patients unfit for surgery include, but are not limited to: inability to tolerate single lung ventilation, severe chronic obstructive pulmonary disease (COPD), and risk of surgery prohibitive. Patients not excluded from surgery will be randomized.

Randomization: Patients will be randomized using a sealed envelope technique. The statistician will generate a randomization order, will print out the ordered treatment assignment documents, and place them in opaque envelopes Subjects enrolled into IPFT Arm will be numbered sequentially started at 101. (e.g. first patient onto Arm A will be patient 101, second patient onto Arm A will be 102). Subjects enrolled onto Surgical Arm will be numbered sequentially starting at 201. (e.g. first patient onto Arm B will be

patient 201, second patient onto Arm A will be 202) No patient identifying information will be documented on the case report forms or in the master study database.

The investigator will open the envelope and reveal the treatment assignment. Within 48 hours of randomization, the primary treatment must be initiated. Patients will be randomized into either initial IPFT or surgery:

B1: IPFT: The IPFT group will receive a total of 5-6 doses of alteplase and DNase delivered through a chest tube or small bore catheter into the pleural space. The doses will be given twice a day. If the first IPFT dose is given in the evening on the first day, they will only receive a total of 5 doses.

OR

B2: Surgery: The surgical arm will have either open surgery or a VATS approach at the discretion of the surgeon.

Protocol Image #2: Chest X-ray PA/Lateral The morning after intervention completion (surgery or last dose of IPFT), a chest X-ray PA/lateral will be obtained (protocol image #2). Based on Image #2 the patient will be categorized into one of three groups: satisfactory improvement of pleural fluid collection, unsatisfactory improvement in pleural fluid collection on imaging, or treatment failures.

To assess radiographic improvement, Image #1 to Image #2 will be evaluated in a standardized, pre-agreed upon fashion by two designated radiologists. Improvement will be broken down into quartiles of improvement (see below).

Radiographic improvement measurement strategy and definitions:

Imaging will be posterior-anterior (PA) and lateral chest x-ray to determine radiographic improvement. Images will be saved and exported as JPEG files and opened in image editing software. Polygons will be drawn representing fluid collections and the hemithorax area covered will be calculated with the volume derived.

Improvement in fluid volume will be quantified as:

- Less than 50%
- Between 50-75%
- Greater than 75%

Improvement of greater than 75% will be considered satisfactory improvement. Chest radiograph images will be reviewed and measured by two independent radiologists. If greater than 5% discrepancy in fluid volume change is reported, the independent radiologists will re-read and re-measure the images together.

If there is **satisfactory improvement** in the pleural fluid collection on imaging **chest tube or small bore catheter will be removed per protocol in both study arms.** Chest tube removal protocol is based on fluid character and measured output.

Algorithm for chest tube removal: Once it has been deemed that there is satisfactory improvement in imaging and no signs of ongoing infection, the chest tube will be removed per protocol. Criteria for removal: Fluid non purulent, serous in character, pleural drain output is less than 200 cc/24 hours.

If protocol image #2 demonstrates **unsatisfactory improvement** in the pleural fluid collection with incomplete drainage or lung re-expansion, then clinical management at the discretion of the physician will occur. This clinical management could include but is not limited to additional imaging, continued observation of the patient with the chest tube or placement of additional targeted pleural drains.

Treatment failures are defined as any patients with evidence of ongoing infection, persistently undrained pleural space and fever or elevated inflammatory markers still present at least 48 hours after completion of their intervention. Treatment failures will be treated at the discretion of the clinically responsible team, this may include but is not limited to study arm crossover. If a patient is a treatment failure and has treatment crossover (IPFT to surgery, surgery to IPFT), the data will documented with the data collection.

Follow-up and Questionnaires: After discharge from the hospital, patients will be seen in outpatient clinic within 14 days +/- 7 days of discharge. Follow-up at 30 days +/- 7 days, and 90 days +/- 7 days and one year +/- 7 days will be via phone for administration of questionnaires (Appendix 2 Phone Script). Quality of life and Work Status will be measured at 30 days, 90 days and 1 year follow-up. Quality of life will be assessed with the SF-36 survey (Appendix 3). Work Status questionnaire will be obtained at baseline (Appendix 4) during the admission then at 30-day via phone (Appendix 5), 90-day via phone (Appendix 6) and via phone at 1 –year (Appendix 7).

Safety Monitoring: There will be regularly scheduled meeting between the research team members including the Principal investigators, research coordinator, and sub-investigators to discuss the study progress and patient results as the study progresses. Both IPFT and surgery are used regularly at Swedish to treat patients with these complicated pleural space infections and have proven efficacy in the treatment of pleural space infections, we are merely trying to compare these two modalities in a randomized setting. Neither approach is experimental.

Cost Comparison: As one of the secondary endpoints for the study, we will perform a cost comparison between the two study arms (IPFT and surgical drainage). Cost data will be obtained from billing data from the patient's hospital admission for stay and procedures related to the patients complicated pleural space infection. No billing data will be obtained directly from the participants in the study.

7.0 Data and Specimen Banking*

The Principal Investigator and research coordinator will maintain an excel spreadsheet of abstracted data on a protected research drive (T drive) only accessible to study investigators. Patient identity will be kept confidential by using assigned subject numbers instead of direct patient identifiers. The spreadsheet will be maintained by the Principal Investigatory and deleted 3 years after the end of the study.

Data will be populated from the clinical data record recorded in either the electronic medical record or on paper forms as applicable. Data will be transferred from the medical record and entered into a study master database. In the event an electronic study master database is unavailable paper case report forms will be completed. The electronic master database will contain the same fields as the paper case report forms. The master database will be password-protected in a secure data storage drive within the Swedish network. No data will be stored on personal devices or USB drives. Only the investigators listed and their designees will have password access to the master file data. A de-identified data set will be extracted from the master list and maintained for use by other members of the research team for the purposes statistical analysis and documentation. When the data is distributed for publication or otherwise disclosed to a source outside of the treatment team, it will be identified by only the assigned subject numbers.

The study may be modified or discontinued at any time by the IRB, OHRP, or other Government agencies as part of their duties to ensure that research subjects are protected. The study investigators will closely monitor study participants for any evidence of unanticipated untoward events. In addition, the research coordinator may provide interim medical chart reviews following the 10th patient completing treatment. The study team, led by the principle investigator will review these data and make the determinations as follows.

The SCI designation for the determination of whether an adverse event is related to treatment will be:

- Related – includes adverse events that are definitely, probably, or possibly related to the medical treatment or procedure;
- Not Related – includes adverse events that are doubtfully related or clearly not related to the medical treatment or procedure.

The SCI Serious Adverse Event (SAE) Report Form will be completed for all serious adverse events that meet the expedited reporting requirements. The SAE form should be faxed to the PI. All available information should be submitted but it is acceptable to fax an incomplete report form at the initial report. A completed report should be faxed as soon as possible but must be received within 15 calendar days.

Serious adverse events that do not meet the requirement for expedited reporting (not related to study treatment or expected) must be reported to the IRB as part of the annual renewal of the protocol.

8.0 Sharing of Results with Subjects*

The results will not be shared directly with subjects; however, if published the paper would be made available to any subject requesting the study.

9.0 Study Timelines*

Accrual of patients will take 12-18 months. Participants will take part in the study for the duration of inpatient clinical care and 1-year following discharge. Primary analysis will occur after the completion of patient accrual.

10.0 Inclusion and Exclusion Criteria*

Inclusion Criteria:

- Age >18 years
- Clinical presentation compatible with pleural infection (fever or leukocytosis, elevated procalcitonin, elevated CRP)
- Pleural fluid requiring drainage that is either:
    - Macroscopically purulent **or**
    - Positive on culture for bacterial infection **or**
    - Positive for bacteria on gram stain **or**
    - LDH > 1000 IU/L **or**
    - Glucose <40 mg/dL

Exclusion Criteria:

- Age <18 years
- Unable to give consent
- Not proficient in English
- History of prior ipsilateral complicated pleural spaceinfection
- Has known sensitivity to DNase or alteplase
- History of intracranial hemorrhage or acute intracranial hemorrhage
- History of stroke, hemorrhage, or trauma within the last 3 months
- Has had prior surgery on the side of the pleural infection
- Patients who are pregnant or lactating
- Expected survival less than 6 months from a different pathology to this pleural infection based on clinical judgment
- Has a tunneled pleural catheter in place
- Patients on anticoagulation that cannot be interrupted for surgical intervention
- Patients with known or suspected malignant pleural effusion
- Patients with renal failure (CrCl <30)

- Prior history of or concern for chylothorax or pseudochylothorax
- Prisoners
- Hemothorax
- Kaiser-Permanente Patients (Due to contracting barriers)
- Evidence of clinically significant bilateral effusions at time of evaluation
- Intrapleural fibrinolytic therapy given prior to study screening

Evidence of clinically significant bilateral effusions at time of evaluation All inpatients admitted to Swedish Medical Center First Hill campus are eligible to be screened for participation. Medical Hospitalist, Intensivists, and Thoracic Surgery services are the primary services responsible for hospital admissions for patients with pleural space processes. Overnight and daytime teams will be contacted daily to identify patients appropriate for screening. Members of the Kaiser-Permanente health care plan will be excluded due to contracting barriers.

Patients who meet all inclusion criteria and have no exclusion criteria are eligible for screening. A designated member of the clinical team will approach participants who fulfil the criteria for inclusion into the trial. Study personnel will perform all necessary procedures and evaluations to document that the subject meets each eligibility criterion Blood samples for hematology and serum chemistry and additional testing will collected at Screening and evaluated by a local laboratory. In addition, they will collect and record the subject's complete history including concurrent medical signs and symptoms. Disease history, including the date of initial diagnosis and prior treatment therapies will also be recorded. Any condition prior to first dose will be treated as medical history.

11.0 Vulnerable Populations*

The study will not include any vulnerable populations.

12.0 Local Number of Subjects

As this is a pilot study to test for feasibility of the study algorithm, we will aim for a total of 30 patients, with the goal of randomizing 20 patients with 10 patients in each study arm (IPFT vs surgical debridement/decortication). We would like to take into account those patients who are not randomized either due to their pleural fluid draining completely after chest tube placement or those that are deemed ineligible for surgery as we will be collecting their data despite them not being randomized. Thus total accrual of subjects is adjusted to 30 patients to ensure that 20 will be available for analysis after randomization. Consecutive patients meeting criteria will be enrolled and randomized using a sealed envelope system.

13.0 Recruitment Methods

Patients will be recruited from inpatient hospital admissions. It will be clearly indicated to the patient that their decision to participate or not will have no impact on their care.

The inpatient hospital admission teams will be educated on the study and inclusion criteria. The overnight and daytime teams will be contacted daily by the study coordinator to identify potential patients for screening. In addition, consults to the thoracic surgeons or interventional pulmonologists for management of pleural space infections will be screened. Screening will consist of the coordinator reviewing records to see if patient meets the inclusion criteria. All participants identified with proven complex pleural space infections fulfilling the inclusion criteria are suitable for randomization. The principle investigator and sub-investigators will have oversight of eligibility with the screened patients reviewed with the investigators.

There will not be any print documents or advertisements used to recruit subjects. There is no subject reimbursement for participating in this study.

14.0 Withdrawal of Subjects*

There are no anticipated circumstances in which subjects will be withdrawn from the research without their consent. Patients who are randomized to the surgery arm and then do not have surgery will be followed and data will continue to be collected. They will not be withdrawn from the study. Similarly if patients randomized to the IPFT arm do not receive IPFT, they will be followed and data will continue to be collected. If a patient wishes to withdraw from the study, then no further data will be collected on the patient. If a patient withdraws, they will be replaced in the study by another patient in order to keep the sample size approximately 30.

15.0 Risks to Subjects*

Both IPFT and surgery are used regularly and considered as standard of care nationally as well as at the Swedish Medical Center and Cancer Institute for treatment of complex pleural space infections. The administration of IPFT and surgery carry their own sets of risks independent of being enrolled in the study. Risks associated with IPFT include intrapleural hemorrhage, hemoptysis, pain, erythema or rash. Surgical risks include bleeding, risk of damage to surrounding structures as well as the risks associated with anesthesia. The placement of a chest tube or pigtail also has risks associated with it including pneumothorax and bleeding. Patients would have a chest tube or pigtail placed for management of their complex pleural space infection regardless of enrollment in the study.

Since both regimens are considered standards of care, it is not anticipated that patients will be exposed to research-specific risks other than the potential loss of the fidelity of medical data. In order to minimize these risks, de-identified, limited data sets

will be utilized to communicate study data whenever possible. In these data sets, patients will be only identifiable by an assigned study number. Only the master database will correlate patient identities to assigned study numbers.

16.0 Potential Benefits to Subjects*

There are no direct patient benefits from enrolling in this study. However there are potential future benefits to future patients with complex pleural space infections as we compare IPFT to surgery for treatment. The comparative effectiveness of IPFT and surgical drainage is unknown.

17.0 Data Management* and Confidentiality

Abstracted data will be saved on an excel spreadsheet and saved on the secured Swedish Medical Center network drive. The spreadsheet will include a sequential number auto-generated by the designed database query. This number (i.e. AutoNumber) will be the only link to the subject's identity (AutoNumber, name, MRN and dates stored on a separate database) should the investigators need to go back to their medical records. There will be no other direct patient identifiers, only subject numbers (AutoNumber) will be assigned.

Any unanticipated adverse events will be promptly reported to the Swedish IRB within 10 working days of the occurrence (or discovery of). Upon the determination of the Board, further notification (e.g. subject, appropriate regulatory authorities) may be required.

Statistics:

Outcome measures related to feasibility of the algorithm, as well as subject identification and accrual will be summarized and reported; while clinical outcomes between the two treatment arms will be compared. Categorical data will be summarized as frequencies and percentages and when compared, a Chi-squared test will be used. Continuous data will be summarized as mean/standard deviation and compared using the student t-test, or median/interquartile range and compared using Mann-Whitney U and Kruskal-Wallis tests, if not normally distributed.

18.0 Provisions to Monitor the Data to Ensure the Safety of Subjects*

Patients will be randomized into one of two modalities that are already used as standard of care at Swedish Medical Center and Cancer Institute for the treatment of complex pleural space infections. When any serious adverse event occurs the PIs will be made aware. An adverse event is considered to be a notable adverse event when an event meets the regulatory criteria for seriousness but which is expected in the study population. The decision regarding whether the adverse event or death is attributable to the natural history of the pleural infection is a clinical judgment of the PI. All serious and notable events will be reviewed by the

PIs and sub-investigators at our research meeting as the study is in progress in case report forms and reported to regulatory and IRB if meets the reporting requirements. Adverse events must be reported within 10 working days and serious adverse events must be reported within 24 hours.

After a total of 10 patients have been enrolled in the study, a preliminary data analysis will be collected to examine both the benefits and potential harms of the study. Data reviewed will include any complications of IPFT or surgery, length of hospitalization, length of chest tube duration, treatment failures, and mortalities.

Adverse events Monitoring and Collection

All subjects will be monitored for AEs during the study. AE grading will be done using CTCAE 4.03 when applicable. Events that occur following the first dose of medication will be treated as an AE. Assessments may include monitoring of any or all of the following parameters: the subject's clinical symptoms, laboratory, pathological, radiological or surgical findings, physical examination findings, or findings from other tests and/or procedures. Data collected will include those risks associated with IPFT include intrapleural hemorrhage, hemoptysis, pain, erythema or rash. Surgical risks include bleeding, risk of damage to surrounding structures as well as the risks associated with anesthesia.

Provisions to Monitor the Data to Ensure the Safety of Subjects

Definitions

1. Adverse Event - Any untoward medical occurrence in a patient or clinical investigation subject administered a pharmaceutical product, medical treatment or procedure and which does not necessarily have to have a causal relationship with this treatment. An adverse event can therefore be any unfavorable and unintended sign (including an abnormal laboratory finding, for example), symptom, or disease temporally associated with the use of a medicinal product, medical treatment or procedure whether or not considered related to the medicinal product.
2. Life-threatening Adverse Event – Any adverse event that places the patient or subject, in view of the investigator, at immediate risk of death from the reaction.
3. Unexpected Adverse Event – An adverse event, the nature or severity of which is not consistent with the applicable product information (e.g., Investigator's Brochure for an unapproved investigational product or package insert/summary of product characteristics for an approved product). If applicable product information is not available, such as for studies that do not involve pharmaceutical products or devices, an unexpected adverse event is an adverse event that was not described in the study protocol or informed consent.
4. Serious Adverse Event (SAE) – Any adverse event occurring that results in any of the following outcomes:
    - death;
    - a life-threatening adverse event (real risk of dying);
    - a persistent or significant disability/incapacity;
    - a congenital anomaly;

- requires intervention to prevent permanent impairment of damage (for example)
- cardiac arrest requiring resuscitation, intubation for respiratory distress, dialysis for renal failure)

19.0 Provisions to Protect the Privacy Interests of Subjects

Patients with complex pleural space infections and empyema will interact with the same medical personnel for management of their disease regardless of if they are enrolled in this study or not. This includes hospitalists, intensivists, members from the Division of Thoracic Surgery and Interventional Pulmonology.

The only additional member they will interact with is the research coordinator during the consent process, follow up visit to complete the SF-36 questionnaire, and at other time points during the study as needed

The research team will have access to the patient's medical records in order to gather the data for the study. Abstracted data will be saved on an excel spreadsheet and saved on the secured Swedish Medical Center network drive. The spreadsheet will include a sequential number auto-generated by the designed database query. This number (i.e. AutoNumber) will be the only link to the subject's identity (AutoNumber, name, MRN and dates stored on a separate database) should the investigators need to go back to their medical records. There will be no other direct patient identifiers, only subject numbers (AutoNumber) will be assigned

20.0 Compensation for Research-Related Injury

All treatments, testing and interventions will be performed as typical to standard of care. Therefore, no research related injury is anticipated.

There is no commitment by Swedish Medical Center, Swedish Health Services, or the study doctors to provide monetary compensation or free medical care in the event of a study-related injury. The charges for such care will be billed to the patient and/or their insurance company which may include co-pays and deductibles.

21.0 Economic Burden to Subjects

There will be no economic burden to subjects enrolled in the study. All procedures in this study will be billed as standard of care.

22.0 Consent Process

Consecutive patients with a pleural infection admitted to the Swedish Cancer Institute will be screened to determine eligibility for inclusion in the study. The principal investigator or a trained and delegated member of the research staff will approach

participants who fulfil the criteria for inclusion into the trial.

A blank consent form (attached) will then be given to the participant; the doctor/trial team will then discuss the study aims, design and potential risks involved. The consent process will take place after the patient has been identified and is admitted to Swedish Medical Center. The patients will be given the opportunity to review the consent and discuss with whomever they wish while the consenting staff are not present, then will be asked to express their understanding of the study, and whether they wish to participate. Patients will need to sign consent at that time if they agree to participation in the research study.

Patients unable to consent and non-English speaking patients will be excluded from the study.

23.0 Process to Document Consent in Writing

We will be following the "SOP: Written Documentation of Consent (HRP-091)."

24.0 Setting

All research will be conducted at Swedish Medical Center First Hill including patient selection, consent, randomization, and treatment. Follow up after discharge will occur in the thoracic clinic in the offices of the Division of Thoracic Surgery, 1101 Madison Street, Suite 900, Seattle, WA, 98104.

25.0 Resources Available

The study will be performed at Swedish Medical Center through the Division of Thoracic Surgery and Interventional Pulmonology. The Division includes 5 Thoracic Surgeons and 2 Interventional Pulmonologists. This division organization combines the support and resources of both the medical and surgical specialties key to this trial. With the specialty cooperation already established for the participation and strategy of this trial, the algorithm methodology and analysis can be closely evaluated to determine the feasibility of the trial logistics and endpoints.

Groundwork for this study was performed by looking retrospectively at recent institutional data for patients with complicated parapneumonic effusions and empyema treated with either IPFT or surgery from 2013-2017, we treated approximately 50-60 patients a year with either IPFT or surgery. Our retrospective study suggests that we have the adequate population of patients to enroll in this pilot study.

Accrual of patients will take 12-18 months. Participants will take part in the study for the duration of inpatient clinical care and 90 days following discharge.

26.0 Multi-Site Research*

N/A. This is a single-center research study.

References:

1. Ferguson a D, Prescott RJ, Selkon JB, Watson D, Swinburn CR. The clinical course and management of thoracic empyema. *QJM*. 1996;89(4):285-289. doi:10.1093/qjmed/89.4.285.

2. Light RW. Parapneumonic Effusions and Empyema. *Proc Am Thorac Soc*. 2006. doi:10.1513/pats.200510-113JH.

3. Wait MA, Sharma S, Hohn J, Dal Nogare A. A randomized trial of empyema therapy. *Chest*. 1997. doi:10.1378/chest.111.6.1548.

4. Colice GL, Curtis A, Deslauriers J, et al. Medical and surgical treatment of parapneumonic effusions: An evidence-based guideline. *Chest*. 2000. doi:10.1378/chest.118.4.1158.

5. Group GW, Shen KR. 2015 AATS Guidelines for Management of Empyema. 2015.

6. Davies H, Davies R, Davies C. BTS Pleural Disease Guideline 2010 British Thoracic Society Pleural Disease Guideline Group Thorax AN INTERNATIONAL JOURNAL OF RESPIRATORY MEDICINE. *Thorax*. 2010;65(Suppl II).

7. Rahman NM, Phil D, Maskell NA, et al. Intrapleural Use of Tissue Plasminogen Activator and DNase in Pleural Infection. 2011;6(11).
