# Supplementary material for: Effect of Intrapleural Fibrinolytic Therapy vs Surgery for Complicated Pleural Infections: A Randomized Clinical Trial
Source: JAMA Netw Open. 2023 Apr 12;6(4):e237799. doi: 10.1001/jamanetworkopen.2023.7799 (PMC10098968; doi:10.1001/jamanetworkopen.2023.7799)
Supplement: Supplement 2. — eTable 1. Exclusion Criteria eTable 2. Microbiology Identified in Pleural Infections With a Positive Pleural Fluid Culture for the Total Population (N = 26) [file jamanetwopen-e237799-s002.pdf]

Supplementary Online Content

Wilshire CL, Jackson AS, Vallières E, et al. Effect of intrapleural fibrinolytic therapy vs surgery for complicated pleural infections: a randomized clinical trial. *JAMA Netw Open*. 2023;6(4):e237799. doi:10.1001/jamanetworkopen.2023.7799

eTable 1. Exclusion Criteria

eTable 2. Microbiology Identified in Pleural Infections With a Positive Pleural Fluid Culture for the Total Population (N = 26)

This supplementary material has been provided by the authors to give readers additional information about their work.

© 2023 Wilshire CL et al. *JAMA Network Open*.

eTable 1. Exclusion Criteria

1. Age <18 years
2. Unable/refused to give consent
3. Not proficient in English
4. History of prior ipsilateral complicated pleural infection
5. Known sensitivity to DNase or alteplase
6. History of acute intracranial hemorrhage
7. History of stroke, hemorrhage, or trauma within the last 3 months
8. Prior ipsilateral surgery
9. Pregnant or lactating
10. Expected survival <6 months from a different pathology based on clinical judgment
11. Tunneled pleural catheter in place
12. On anticoagulation that cannot be interrupted for surgical intervention
13. Known or suspected malignant pleural effusion
14. Renal failure
15. Prior history of or concern for chylothorax or pseudochylothorax
16. Vulnerable populations: prisoners
17. Hemothorax
18. Kaiser-Permanente insurance (due to contracting barriers)
19. Evidence of clinically significant bilateral effusions at time of evaluation
20. Intrapleural fibrinolytic therapy given prior to study screening

© 2023 Wilshire CL et al. *JAMA Network Open*.

eTable 2. Microbiology Identified in Pleural Infections With a Positive Pleural Fluid Culture for the Total Population (N = 26)

	**Number (%)**
Streptococcus intermedius	5 (19%)
Methicillin-resistant Staphylococcus aureus	3 (12%)
Streptococcus pneumoniae	2 (7%)
Beta Hemolytic Streptococci Group F	1 (4%)
Staphylococcus aureus	1 (4%)
Staphylococcus epidermidis	1 (4%)
Streptococcus constellatus	1 (4%)
Streptococcus constellatus/intermedius	1 (4%)
Streptococcus anginosus/intermedius	1 (4%)

© 2023 Wilshire CL et al. *JAMA Network Open*.
