# Supplementary material for: Effect of Intrapleural Fibrinolytic Therapy vs Surgery for Complicated Pleural Infections: A Randomized Clinical Trial
Source: JAMA Netw Open. 2023 Apr 12;6(4):e237799. doi: 10.1001/jamanetworkopen.2023.7799 (PMC10098968; doi:10.1001/jamanetworkopen.2023.7799)
Supplement: Supplement 3. — Data Sharing Statement [file jamanetwopen-e237799-s003.pdf]

Data Sharing Statement

Wilshire. Effect of Intrapleural Fibrinolytic Therapy vs Surgery for Complicated Pleural Infections. *JAMA Netw Open*. Published April 12, 2023.
doi:10.1001/jamanetworkopen.2023.7799

Data
Data available: Yes
Data types: Deidentified participant data
How to access data: Data will be made available upon request: candice.wilshire@swedish.org.
When available: With publication

Supporting Documents
Document types: Other (please specify)
Additional Information: Trial protocol.
How to access documents: Data will be made available upon request: candice.wilshire@swedish.org.
When available: With publication

Additional Information
Who can access the data: Researchers whose proposed use of the data has been approved.
Types of analyses: Research purposes.
Mechanisms of data availability: After approval of a proposal, or with a signed data access agreement
